# Antimalarial drug and renal toxicity

**Published:** 2015-12-23

**Authors:** Viroj Wiwanitkit

**Affiliations:** Hainan Medical University, Hainan, China

**Keywords:** Malaria, Kidney, Toxicity, Chloroquine

**Implication for health policy/practice/research/medical education:**

Malaria is a common tropical infection. This infection is still a big global health problem. Treatment of the infected cases requires antimalarial drug administration. Similar to any drug, the adverse effect of antimalarial drug can be seen. The renal toxicity of antimalarial drug is considered rare. The concern is on the use of the drug in the case with underlying renal problem.

## Introduction

Malaria is a common tropical infection. It is a kind of mosquito borne infection. The pathogen is the protozoa in Plasmodium spp. The patient usually gets acute febrile illness after get mosquito bite at malarial area. The infection can still be seen in several countries. The cases can be presently seen not only in tropical but non-tropical countries due to good transportation system. This infection is still a big global health problem. Early diagnosis and treatment is the key activity for management of the patient. Treatment of the infected cases requires antimalarial drug administration. Similar to any drug, the adverse effect of antimalarial drug can be seen. Here, the author briefly reviews and discusses on antimalarial drug and renal toxicity (1).

## Chloroquine and renal toxicity

Chloroquine is a classical antimalarial drug. It seems to be out-of-date at present due to the drug resistance problem. Focusing on the renal toxicity of chloroquine, there is no clear evidence in administration for management of malaria. However, in long-term use, which is not the case of malaria, the problem of renal toxicity be seen (1,2). There are also some concerns on the use of chloroquine in the patients with concomitant renal problem. Thorogood et al noted that the severe acute megaloblastic anemia, exfoliative dermatitis and symptomatic pancytopenia could be a serious complication in patients with underlying end- stage kidney failure who got chloroquine (3).

## Quinine and renal toxicity

Quinine is an effective antimalarial drug. The allergy to this drug is possible (4,5) and renal problem can be seen. In case with allergy, neutropenia, anemia, thrombocytopenia, disseminated intravascular coagulation, acute renal failure, liver toxicity and neurological abnormalities can be seen (4).

The concern on using quinine in the cases with renal problem should also be mentioned. Padmaja et al concluded that quinine should be used cautiously in patients with impaired kidney function (6). Franke et al mentioned that in state of acute kidney injury in cerebral malaria, the dose of quinine should be diminished, however, the common recommendation of 10 to 15 mg/kg^-1^/day^-1^ may be too low, and that hemofiltration has no marked influence on the total body clearance of quinine (7).

## Artesunate and renal toxicity

Artesunate is the new antimalarial drug. It is proved for effectiveness and there is no problem of drug resistance. Also, it is applied as alternative additional therapy for cancer (8). Focusing on renal toxicity of artesunate there are some reports. On rat model, it is shown that reversible nephrotoxicity could be seen (9). Campos et al found that artesunate diminishes glomerular filtration rate and increases kidney blood flow and urinary excretion of Na, Cl, and K (10). However, it is still not the problem for short-term low dosage use in treatment of malaria. Although there are some case reports on renal failure after malarial treatment with artesunate, it is proved for no causal relationship with artesunate (11). In addition, using artesunate with homeopathic medicine in China showed no effect on renal function (12). Additionally, there is also an animal experimental study showing the clinical usefulness of artesunate in management of nephrotic syndrome (13).

## Conclusion

The renal toxicity of antimalarial drug is considered rare. The concern is on the use of the drug in the case with underlying renal problem.

## Author’s contribution

VW is the single author of the paper.

## Conflicts of interest

The author declared no competing interests.

## Ethical considerations

Ethical issues (including plagiarism, data fabrication, double publication) have been completely observed by the author.

## Funding/Support

None.
